# Tophaceous Gout and Renal Insufficiency: A New Solution for an Old Therapeutic Dilemma

**DOI:** 10.1155/2011/397646

**Published:** 2011-05-14

**Authors:** Anne-Kathrin Tausche, Carsten Wunderlich, Martin Aringer

**Affiliations:** ^1^Section of Rheumatology, Department of Internal Medicine, University Clinic “Carl Gustav Carus” of the Technical University of Dresden, Fetscherstrasse 74, 01307 Dresden, Germany; ^2^Department of Cardiology and Intensive Care, Heart Centre Dresden, University Hospital of the Technical University of Dresden, Fetscherstrasse 74, 01307 Dresden, Germany

## Abstract

The prevalence of gout is increasing with increased life expectancy. Approximately half of the patients with gout have some degree of renal impairment. If both conditions persistently coexist, and in severe tophaceous gout, in particular, treatment has been difficult. We here report on the case of an 87-year-old woman, who had been suffering from recurrent gouty arthritis over 4 years. Monthly polyarthritis attacks were accompanied by subcutaneous tophi. Serum uric acid levels were constantly above 600 **μ**mol/L (10 mg/dL). Allopurinol was no option because of intolerance, while benzbromarone was ineffective because of renal impairment. Therefore, the novel xanthin oxidase inhibitor febuxostat was started, achieving rapid control of serum urate levels (<360 **μ**mol/L). After initial worsening of inflammation in the first weeks, gouty attacks stopped and all tophi resolved within the following 10 months. Renal function remained stable.

## 1. Introduction

Hyperuricemia, defined as a serum urate concentration higher than the limit of urate solubility (408 *μ*mol/L or 6.8 mg/dL at 37°C, pH 7.4), is common and can manifest clinically as the urate crystal deposition disease, gout [1]. Gout is the most common arthritis in men, and its incidence is rising with age [2]. If the underlying hyperuricemia remains without sufficient therapy for years, chronic tophaceous gout may manifest. In addition to other comorbidities, such as hypertension, diabetes, and cardiovascular disease, approximately every second patient with gout has some degree of renal impairment, defined by an estimated glomerular filtration rate (eGFR) of less than 90 mL/min per 1.73 m^2^ as per Cockroft-Gault equation corrected for ideal body weight [3].

## 2. Case Presentation

An 87-year-old woman was seen at the rheumatology because of chronic tophaceous gout. Monthly polyarticular gout flares afflicted both first metatarsophalangeal joints, as well as both wrists and multiple finger joints for approximately 4 years. The patient became bed-ridden, could not perform simple, everyday tasks of self-care (e.g., washing, dressing) and daily living (e.g., laundry, cooking), and required help from her daughter. 

Two years earlier, her general practitioner had appropriately started allopurinol. However, after 4 weeks, the patient had developed an itching generalised exanthema, so that allopurinol had to be stopped. Benzbromarone was given at a dose of 50 mg q.d. but had an insufficient effect on serum uric acid levels, which still remained at 500 *μ*mol/L. During the last year, the patient had additionally developed multiple tophi located over the distal finger joints and both metatarsophangeal joints. Her pre-existing renal insufficiency had worsened (eGFR 30 mL/min). Further comorbidities of the patient included arterial hypertension, noninsulin-dependent diabetes mellitus, and permanent atrial fibrillation.

**Figure 1:**
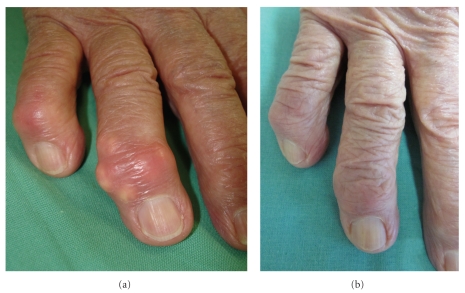
Clinical presentation: Tophi over the right distal interphalangeal joint II. (a) Before therapy with febuxostat. (b) After 10 months of therapy.

**Figure 2:**
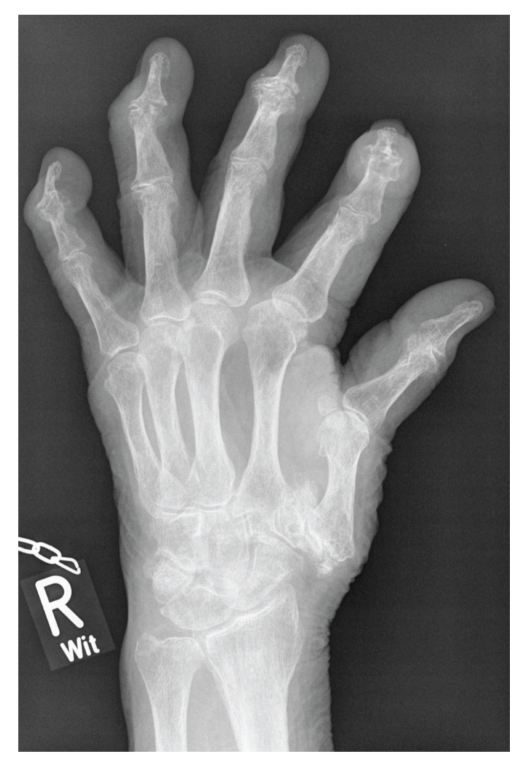
Radiograph of right hand showing the destructive character of recurrent crystal induced arthritis.

On the first admission to our department the patient was sitting in a wheelchair, appeared depressive, and suffered from severe pain (Visual analogue scale, VAS 8-9) and loss of function of distal finger joints, wrists and feet. Physical examination showed normal temperature, elevated blood pressure 160/80 mm Hg and an irregular pulse of 80–90 beats per minute. Otherwise, heart and lungs were normal, as were abdomen and the lumbar region. Joint examination revealed swollen painful wrists and distal finger joints with deformities and loss of mobility. Multiple subcutaneous tophi were visible (Figure 1(a)). Radiographs of both hands and feet showed tophaceous gout and destruction of multiple finger joints (Figure 2). RBC, platelets, LFTs, LDH, protein-electrophoresis, and TSH were unremarkable. Leukocytosis (12.6 GPt/L), elevated CRP (25 mg/L), and serum urate (717 *μ*mol/L) were consistent with chronic gout. Her serum-creatinine (218 *μ*mol/L) and urea (25.2 mmol/L) were increased approximately threefold. The estimated glomerular filtration rate (eGFR) was calculated at 30 mL/min per 1.73 m^2^. 

In this elderly lady with chronic tophaceous gout, significant renal impairment, and other comorbidities, we started urate lowering therapy with 80 mg of febuxostat q.d. (lower doses are not available in Germany). Concomitantly, prophylactic medication for expected gout attacks was started with 10 mg of Prednisolon q.d., 0.5 mg of colchicine e.o.d. day, and low-dose ibuprofen as needed. The patient was advised to carefully monitor daily fluid intake and urinary excretion. As early as one week after the first administration of febuxostat, serum urate level was reduced to 328 *μ*mol/L. Not surprisingly, given this steep decline in serum urate, the patient experienced another severe polyarticular gout flare. Tophi were visibly inflamed, and her eGFR decreased to 20 mL/min. Under forced diuresis and analgesia including narcotics, the situation stabilized within the next 4 weeks; the eGFR improved to 30 mL/min. After 5 months, the gouty attacks have stopped and the tophi have started to resolve (Figure 1(b)). Quality of life improved impressingly (pain on VAS 2-3), and the patient regained the ability to walk without help for up to half an hour.

## 3. Discussion

Coexistence of chronic tophaceous gout and renal insufficiency poses a significant therapeutic challenge. Management of chronic gout is targeted on lowering and maintaining serum uric acid at subsaturating concentrations below 360 *μ*mol/L (6 mg/dL) [4]. The two available pharmacological targets of urate reduction are xanthin oxidase (XO) inhibition and enhancement of urinary excretion with uricosuric agents. However, uricosuric agents, such as benzbromarone and probenecid, have limited effectiveness, and in patients with impaired renal function in particular [5]. Therefore, for decades, the purine analogue XO inhibitor urate has remained the mainstay of gout therapy [6]. Since oxipurinol, its (active) metabolite, is renally excreted, allopurinol requires dose reduction in patients with renal impairment [7]. Such lower doses often are not sufficiently effective. In addition, allergic reactions to allopurinol are not uncommon, and may be severe [8]. In these cases, the nonpurine xanthin oxidase inhibitor febuxostat represents an alternative, because it is chemically distinct and metabolized by the liver and excreted via both liver and kidneys. In general febuxostat is well tolerated in clinical trials [9, 10]. However, some concerns were raised regarding elevation of liver enzymes in 3–5% of patients. Because of a nonsignificant but higher rate of death and cardiovascular events on febuxostat compared to allopurinol 300 mg caution is recommended in patients with ischaemic heart disease or severe congestive heart failure [9, 11]. Febuxostat is approved in Europe, where tablets of 80 and 120 mg febuxostat are available. 

Any urate lowering therapy should not be started during an acute attack, since the dissolution of crystal deposits increases the risk of gout attacks. For the same reason the therapy should be started at a low-dose and titrated up to the effective dose to avoid the “crash”—reduction of uric acid [12]. In our patient, efficacy of febuxostat was somewhat underestimated, and half of 80 mg daily might have been a more appropriate dose, with a lesser risk of severe flares. Appropriate prophylaxis against flares poses the next challenge. Both the use of colchicin and of nonsteroidal anti-inflammatory drugs is severely restricted in such cases, given significant risks. Accordingly, combinations of steroids and narcotics for short time often constitute the best available option today [13].

## 4. Conclusion

We here present the case of an elderly lady with severe polyarticular, tophaceous gout, coexistent renal insufficiency, and allopurinol hypersensitivity. Under therapy with the novel xanthin oxidase inhibitor febuxostat, serum uric acid levels decreased to normal values. Over time, gout attacks stopped and her tophi resolved. Renal function remained stable.

##  Conflict of Interest Statement

All authors verify the mutual agreement to submit this manuscript to the journal for review and declare that this work has not been submitted to any other journals for consideration. Anne-Kathrin Tausche and Martin Aringer have served for Berlin Chemie-Menarini (advisory boards and given lectures). Carsten Wunderlich declares that there is no potential conflict of interest with regards to this manuscript. The final manuscript was reviewed and approved by all authors.
